# Per‐oral endoscopic myotomy as treatment for Killian–Jamieson diverticulum

**DOI:** 10.1002/deo2.27

**Published:** 2021-08-24

**Authors:** Yuto Shimamura, Mary Raina Angeli Fujiyoshi, Yusuke Fujiyoshi, Yohei Nishikawa, Masashi Ono, Kaori Owada, Haruo Ikeda, Manabu Onimaru, Haruhiro Inoue

**Affiliations:** ^1^ Digestive Diseases Center Showa University Koto Toyosu Hospital Tokyo Japan

**Keywords:** esophageal diseases, esophageal diverticulosis, per‐oral endoscopic myotomy

## Abstract

Killian–Jamieson diverticulum (KJD) is a rare type of esophageal diverticulum less commonly encountered compared with Zenker's diverticulum (ZD). Endoscopic approach for these diverticula has been rapidly evolving. Currently, a flexible endoscopic septum division is considered the first‐line treatment for symptomatic ZD patients, however reported recurrence rates are over 10% according to recent literature. With the advent of submucosal tunneling technique established by per‐oral endoscopic myotomy for achalasia, it has been applied to treat ZD named as Zenker's diverticulum per‐oral endoscopic myotomy (Z‐POEM) as a minimally invasive treatment. Although there are very few reports utilizing submucosal tunneling approach to KJD, we have opted to perform Z‐POEM in order to safely perform complete dissection of the muscle septum while maintaining mucosal integration. Due to the difficulty of anatomical location of KJD, we created mucosal incision and subsequent submucosal tunnel directly at the level of the septum as opposed to creating a submucosal tunnel few centimeters proximal to the septum as being previously proposed. We report a case in which this technique was successfully performed with complete resolution of dysphagia without any adverse event. This technique permits to perform complete myotomies without the fear of causing perforation. Although larger cohorts are required to assess its safety and efficacy, Z‐POEM to treat KJD seems to be promising.

## INTRODUCTION

**FIGURE 1:**
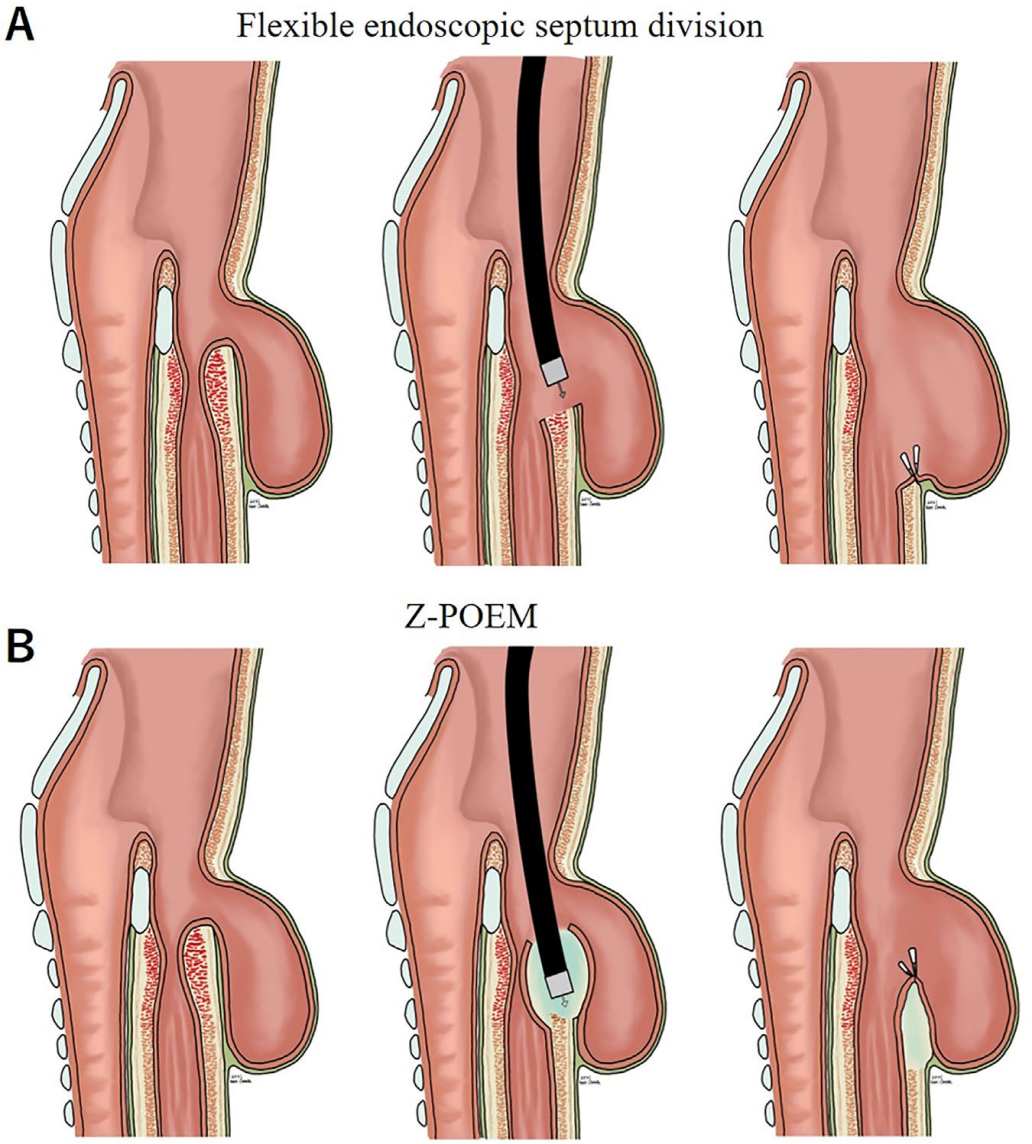
(A) Schematic images of conventional flexible endoscopic septum division. Full thickness incision of the mucosa, submucosa, and the muscular fibers is performed to create a common cavity between the esophagus and diverticulum. (B) Schematic images of Z‐POEM (Zenker's diverticulum per‐oral endoscopic myotomy). A minimal mucosal incision is made in order to advance the endoscope into the submucosal space of the septum. Complete septotomy is performed and mucosal incision site is securely closed with several endoclips

Killian–Jamieson diverticulum (KJD) is a rare type of esophageal diverticulum less commonly encountered compared with Zenker's diverticulum (ZD).^1^ Endoscopic approach for ZD has been widely accepted owing to its safety and efficacy. Currently, flexible endoscopic septum division is considered first‐line treatment for symptomatic Zenker's diverticulum, which involves a full thickness incision of the mucosa, submucosa, and the muscular fibers that form the diverticular septum.^2^ By cutting the entire septum and creating a common cavity between the esophagus and diverticulum, it allows the food content to flow down the true lumen of the esophagus (Figure 1A). The drawback of this technique is that the recurrence rates are over 10% according to recent literature likely due to incomplete septoplasty.^3^ In addition, pooled rate of perforation is reported to be 7% in recent meta‐analysis.^4^ More recently, with the advent of submucosal tunneling technique, Zenker's per‐oral endosocopic myotomy (Z‐POEM) was developed in light of aforementioned result. The risk of perforation, mediastinitis, and recurrence are postulated to decrease as mucosal integrity is maintained even with complete dissection of the muscle fibers (Figure 1B). Theoretically, it also facilitates clear endoscopic visualization of the muscular septum, thus allowing to completely cut the muscle fibers. Recent studies show that Z‐POEM is feasible, safe, and effective in majority of patients.^5^ However, endoscopic approach for KJD has not been established likely due to its rarity of the disease. KJD arises from the anterolateral wall of the cervical esophagus and its sac lies off midline inferior to the cricopharyngeus muscle, which causes limited working space restricting endoscopic maneuverability. Z‐POEM is usually performed by creating the submucosal tunnel from 1 to 2 cm proximal to the septum, however we opted to performed per‐oral endoscopic septotomy allowing direct access to the septum obviating to create a submucosal tunnel. Herein, we report a case of a patient with KJD treated successfully with a per‐oral endoscopic septotomy.

## CASE REPORT

This is a case of a 40‐year‐old Japanese female with no significant past medical history who presented with a few‐year history of solid food dysphagia. Symptoms gradually progressed and started to affect her daily activities. No previous consultation and treatment were noted. The patient underwent a thorough evaluation that includes upper endoscopy, barium esophagogram, and computed tomography (CT) scan of the thorax and upper abdomen.

**FIGURE 2:**
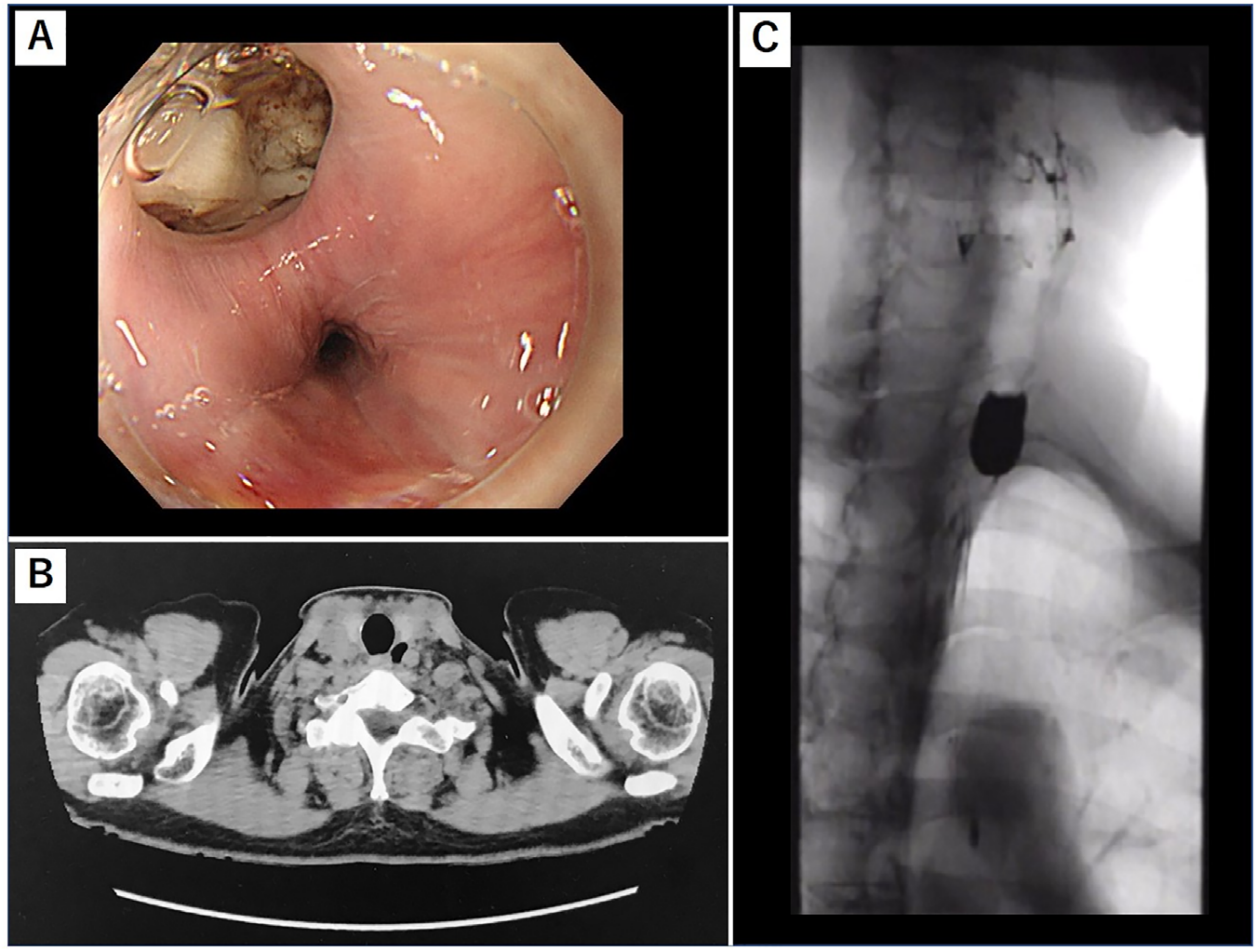
(A) Endoscopic image of Killian–Jamieson diverticulum. Food impaction is seen at 11 o'clock. (B) CT image of Killian–Jamieson diverticulum. The diverticulum lies off midline inferior to the cricopharyngeus muscle and located at the anterolateral wall of the cervical esophagus. (C) Barium esophagram revealing pooling of barium in the diverticulum

Upper endoscopy showed diverticulum located at the anterolateral (11 o'clock position) of the upper esophagus (Figure 2A). Barium esophagogram and CT scan showed outpouching at the level of the upper esophagus findings compatible with KJD (Figure 2B and C). We opted to perform Z‐POEM aiming for complete septotomy. This procedure has been approved by the ethics committee of Showa University Koto Toyosu Hospital and was performed in accordance with the Declaration of Helsinki. Written informed consent was obtained from the patient prior to the procedure.

**FIGURE 3:**
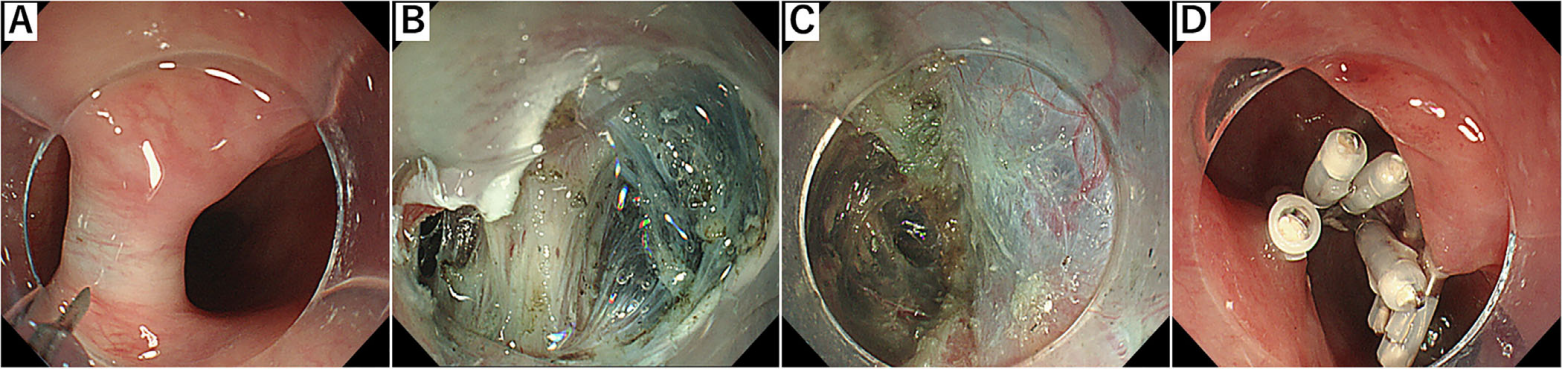
(A) Saline with indigo carmine was injected into the submucosa to create a bleb at the top of the septum and the mucosa was incised. (B) Septotomy was performed under clear endoscopic visualization. (C) Complete septotomy is achieved revealing mediastinum. (D) Mucosal opening was securely closed with endoclips

**FIGURE 4:**
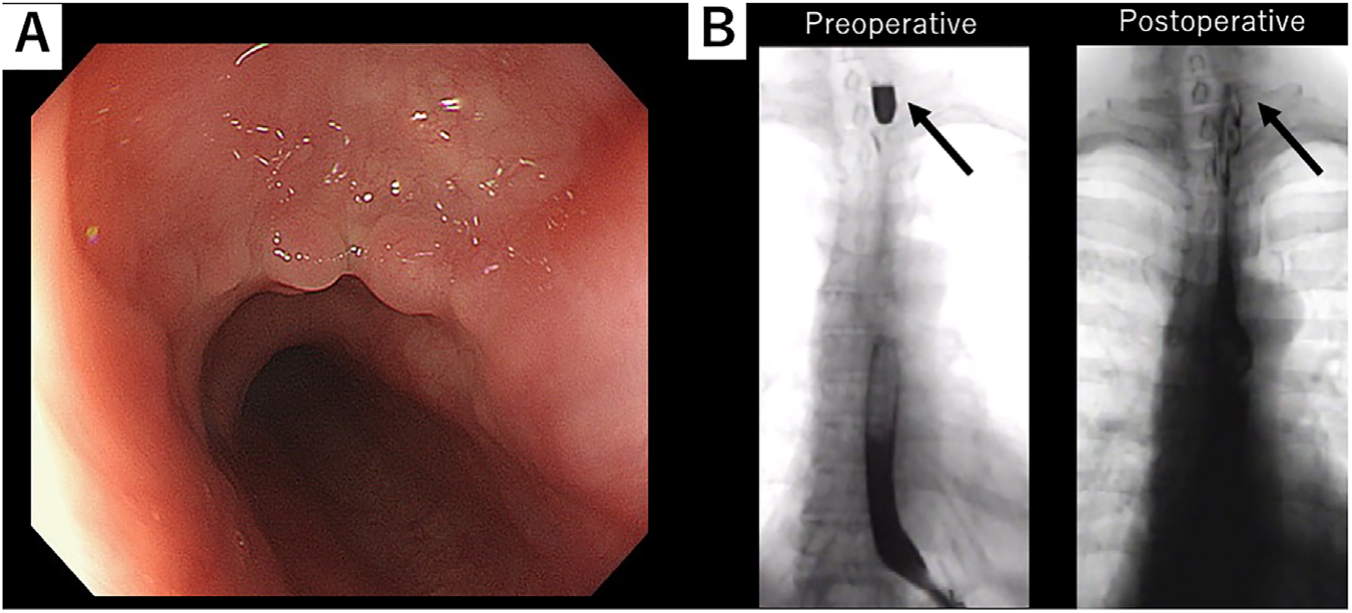
(A) Follow‐up endoscopic image showing complete septotomy creating common cavity between the esophagus and diverticulum. (B) A barium esophagogram showed a marked and sustained improvement in the passage of the contrast

Per‐oral endoscopic septotomy was performed under general anesthesia, with the use of a single‐channel therapeutic endoscope (GIF‐H290T; Olympus Corp., Tokyo, Japan). The super‐soft hood (Space adjuster; TOP Corp., Tokyo, Japan) made of silicon was used as a distal attachment. Food residual was removed, and the diverticulum was confirmed on the left side of the true lumen of the esophagus. Saline with indigo carmine was injected into the submucosa to create a bleb at the top of the septum (Figure 3A). Mucosa was incised with Triangle‐Tip KnifeJ (KD‐645L; Olympus Corp.), which was used throughout the procedure. Submucosal tunnel was created and circular esophageal muscle that constitutes the septum was identified (Figure 3B). This was dissected utilizing spray coagulation under direct endoscopic visualization (Spray coagulation mode, 50 W, effect 2: VIO300D ERBE; Tübingen, Germany). The cutting end of the circular muscle of KJD septum could be directly confirmed by noting the disappearance of muscle fiber (Figure 3C). In this case, the length of myotomy was estimated to be approximately 2 cm, which was compatible with preoperative barium finding. Subsequently, mucosal entry was securely closed with endoclips (Figure 3D). Her diet was advanced after the second look endoscopy and barium esophagogram confirming no leakage on the next day of the procedure. Postoperative clinical course was unremarkable and was discharged home in 3 days after tolerating a soft diet. There was an immediate response of this procedure, with significant improvement in dysphagia. On 2 months follow‐up, the patient remained asymptomatic. A follow‐up upper endoscopy was performed which showed complete septoplasty (Figure 4A) and barium esophagogram showed a marked and sustained improvement in the passage of the contrast (Figure 4B)

## DISCUSSION

KJD is a rare type of lateral pharyngo‐esophageal diverticulum distinct entity different from ZD.^1^ KJD is considered difficult to treat endoscopically due to its anatomical location of the diverticulum but there is infrequent discussion regarding the management. Transcervical surgical myotomy is considered the mainstay approach for the management; however, endoscopic therapies have been reported as safe and effective.^3^ Recently, submucosal tunneling technique (Z‐POEM) was adopted as it has an advantage to completely dissect the septum while maintaining mucosal integrity.^6^, ^7^ Although it has not been proven to be superior to conventional methods, the recent literature shows Z‐POEM is a safe and feasible treatment option with technical success rate of 95% and adverse event rate of 6% comparable with conventional technique.^5^ Endoscopic approach has not been widely applied for KJD as it is technically challenging to create the submucosal tunnel proximal to the septum and to securely close the mucosal defect especially in case with KJD due to limited working space. In order to gain direct access to the muscular septum, we prefer to perform Z‐POEM by cutting mucosa directly on top of the septum, similar to previously proposed per‐oral endoscpic septotomy^7^ and mucosa‐preserving Zenker's diverticulotomy.^8^ In this case, a minimal mucosal incision was performed for the endoscope to advance into the submucosal space of the septum, and complete septum dissection was achieved till reaching the mediastinum. This procedure was done without any technical difficulties and encountering adverse events. Clinical course was uneventful with significant improvement in dysphagia. However, there are some technical cautions. The utmost important part of the procedure is to close the mucosa completely in order to prevent the leakage into the submucosal space. In addition, the risk of recurrent laryngeal nerve injury should be noted. Every step of the procedure should be performed meticulously under direct endoscopic visualization. Midline division of the diverticulum may lower the possibility of nerve injury as the recurrent laryngeal nerve in the proximal tracheoesophageal groove can be either anterior or posterior to the base of KJD.^9^ Also, the extent of myotomy should be well controlled to avoid excessive myotomy potentially lowering the risk of nerve injury.^10^

In summary, Z‐POEM technique seems to be a promising minimally invasive approach theoretically enabling to perform complete septotomy even in tight working space such as in case with KJD. Larger cohorts are required for better understanding to see which patients benefit from this endoscopic approach.

## CONFLICT OF INTEREST

Haruhiro Inoue is an advisor of Olympus Corporation and Top Corporation. He has also received educational grants from Olympus Corp. and Takeda Pharmaceutical Co. All authors have no conflicts of interests to declare pertaining to this report.

## FUNDING INFORMATION

None.

## References

[1] Haddad N , Agarwal P , Levi JR , Tracy JC , Tracy LF . Presentation and management of Killian Jamieson diverticulum: A comprehensive literature review. Ann Otol Rhinol Laryngol 2020; 129:394–400. 31707793 10.1177/0003489419887403

[2] Weusten BLAM , Barret M , Bredenoord AJ , et al. Endoscopic management of gastrointestinal motility disorders—Part 2: European Society of Gastrointestinal Endoscopy (ESGE) Guideline. Endoscopy 2020; 52: 600–14. 32462649 10.1055/a-1171-3174

[3] Ishaq S , Hassan C , Antonello A , *et al*. Flexible endoscopic treatment for Zenker's diverticulum: A systematic review and meta‐analysis. Gastrointest Endosc 2016; 83: 1076–1089.e5. 26802196 10.1016/j.gie.2016.01.039

[4] Li LY , Yang YT , Qu CM , *et al*. Endoscopic needle‐knife treatment for symptomatic esophageal Zenker's diverticulum: A meta‐analysis and systematic review. J Dig Dis 2018; 19: 204–214. 29675866 10.1111/1751-2980.12588

[5] Kamal F , Khan MA , Lee‐Smith W , *et al*. Peroral endoscopic myotomy is a safe and feasible option in management of esophageal diverticula: Systematic review and meta‐analysis. Dig Dis Sci 2020. doi: 10.1007/s10620-020-06678-5 33123940

[6] Maselli R , Spadaccini M , Cappello A , *et al*. Flexible endoscopic treatment for Zenker's diverticulum: From the lumen to the third space. Ann Gastroenterol 2021; 34: 149–54. 33654352 10.20524/aog.2021.0575 PMC7903579

[7] Repici A , Spadaccini M , Belletrutti PJ , *et al*. Peroral endoscopic septotomy for short‐septum Zenker's diverticulum. Endoscopy 2020; 52: 563–8. 32185781 10.1055/a-1127-3304

[8] Zhang LY , Ngamruengphong S . Mucosa‐preserving Zenker's diverticulotomy. VideoGIE 2021; 6: 109‐11. 33738355 10.1016/j.vgie.2020.11.004 PMC7947252

[9] Undavia S , Anand SM , Jacobson AS . Killian‐Jamieson diverticulum: A case for open transcervical excision. Laryngoscope 2013; 123: 414‐7. 23184336 10.1002/lary.23639

[10] Yun PJ , Huang HK , Chang H , Lee SC , Huang TW . Endoscopic diverticulotomy with a stapler can be an effective and safe treatment for Killian‐Jamieson diverticulum. J Thorac Dis 2017; 9: E787‐91. 29221344 10.21037/jtd.2017.08.14 PMC5708513

